# Nonlinear magnetoelectric effect in paraelectric state of Co_4_Nb_2_O_9_ single crystal

**DOI:** 10.1038/s41598-017-14169-3

**Published:** 2017-10-26

**Authors:** Yiming Cao, Guochu Deng, Přemysl Beran, Zhenjie Feng, Baojuan Kang, Jincang Zhang, Nicolas Guiblin, Brahim Dkhil, Wei Ren, Shixun Cao

**Affiliations:** 1 Department of Physics, International Center of Quantum and Molecular Structures, and Materials Genome Institute, Shanghai University, Shanghai, 200444 China; 2 Center for Magnetic Materials and Devices, Key Laboratory for Advanced Functional and Low Dimensional Materials of Yunnan Higher Education Institute, Qujing Normal University, Qujing, 655011 China; 3 Laboratoire Structures, Propriétés et Modélisation des Solides, CentraleSupélec, CNRS-UMR 8580, Université Paris-Saclay, 91192 Gif-sur-Yvette, France; 4 Australian Nuclear Science and Technology Organisation, New Illawarra Road, Lucas Heights, NSW 2234 Australia; 5 Nuclear Physics Institute CAS, 25068 Rez, Czech Republic; 6 Shanghai Key Laboratory of High Temperature Superconductors, Shanghai University, Shanghai, 200444 China

## Abstract

We report the structural, magnetoelectric (ME), magnetic and electric control of magnetic properties in Co_4_Nb_2_O_9_ (CNO) single crystal. A detailed ME measurement reveals a nonlinear ME effect instead of a linear ME effect in CNO single crystal. By fitting the magnetization-electric field (M-E) curve, it can be found that the linear (${\alpha }_{e}$) and quadratic (γ) coefficients equal to ~8.27 ps/m and ~−6.46 ps/MV for upper branch, as well as ~8.38 ps/m and ~6.75 ps/MV for the lower branch. More importantly, a pronounced response was observed under a small cooling magnetic field, which cannot even cause the spin flop. This suggests a magnetoelectric effect can occur at paraelectric state for CNO single crystal. Furthermore, we also found that the magnetization of every axis responds to electric field applied along *a*-axis, but fails to do so when the electric field is applied *c*-axis. Such findings supply a direct evidence to the magnetic structure and ME coupling mechanism indirectly reflected by our neutron experiment.

## Introduction

Multiferroics have attracted a lot of attention as they exhibit coexistence of two or more switchable states such as polarization, magnetization or strain^1,2^ rendering them very interesting for both their intriguing fundamental physics and promising applications. Because weak coupling between magnetic moments and electric polarization is usually observed in single-phase materials^3^, there is an important research effort towards novel materials with improved magnetoelectric coupling. Indeed, highly efficient control of magnetism by means of an electric field^4^ may find a plethora of applications in magnetic storage and magnetic random-access memory devices.

In the route towards materials having strong magnetoelectric (ME) responses, Co_4_Nb_2_O_9_ (CNO) is very attractive. In polycrystalline form, it has been shown to display a large linear ME coupling as well as an electric field control of the magnetism^5^, a manipulation which remains quite rarely reported. According to previous report by Kolodiazhnyi *et al*.^6^, the structural space group of CNO is $P\mathop{3}\limits^{\bar{} }c1$. And a magnetodielectric peak attributed to magnetically-driven spin-flop phase transition was observed in polycrystalline CNO near the antiferromagnetic phase transition temperature (T_N_ = 27.5 K) under a high external magnetic field (>12 kOe). Interestingly, both the magnetically-induced electric polarization and the control of magnetization with an electric field were observed in polycrystalline CNO by Fang *et al*.^5^. Our former work showed that the magnetic moments of CNO are located in *ab*-plane^7^ but not along the *c*-axis. Moreover, Khanh *et al*. proposed a magnetic structure with space group *C2/c’* by using single-crystal neutron diffraction^8^. If the magnetic structure corresponds to that space group, an antiferromagnetic phase transition along *c*-axis should be therefore observed. Surprisingly, there is no antiferromagnetic phase transition in our *c*-axis magnetic measurements^7^, which was also unseen by Khanh *et al*.^8^. In very recent reports, Khanh *et al*.^8^ and Solovyev *et al*.^9^ finally clarified the origin of ME effect in the centrosymmetric trigonal Co_4_Nb_2_O_9_ and Co_4_Ta_2_O_9_. Furthermore, a structural phase transition with *a*-axis shrinkage and *c*-axis expansion at T_N_ was reported by Yin *et al*.^10^.

In order to better understand the magnetic behavior in this material with large ME coupling, we conducted a new neutron diffraction measurement and suggested a mechanism of the ME coupling in the single crystals of CNO^11^. In this work, our detailed ME measurements reveal a nonlinear ME effect instead of a linear one as previously reported in CNO single crystals. More importantly, a pronounced response was observed under a small cooling magnetic field for which it cannot even cause the spin flop transition. Furthermore, we also found that the magnetization of every axis responds to electric field applied along *a*-axis, but unchanged when the electric field is applied *c*-axis.

## Results

### Analysis of neutron diffraction

**Figure 1:**
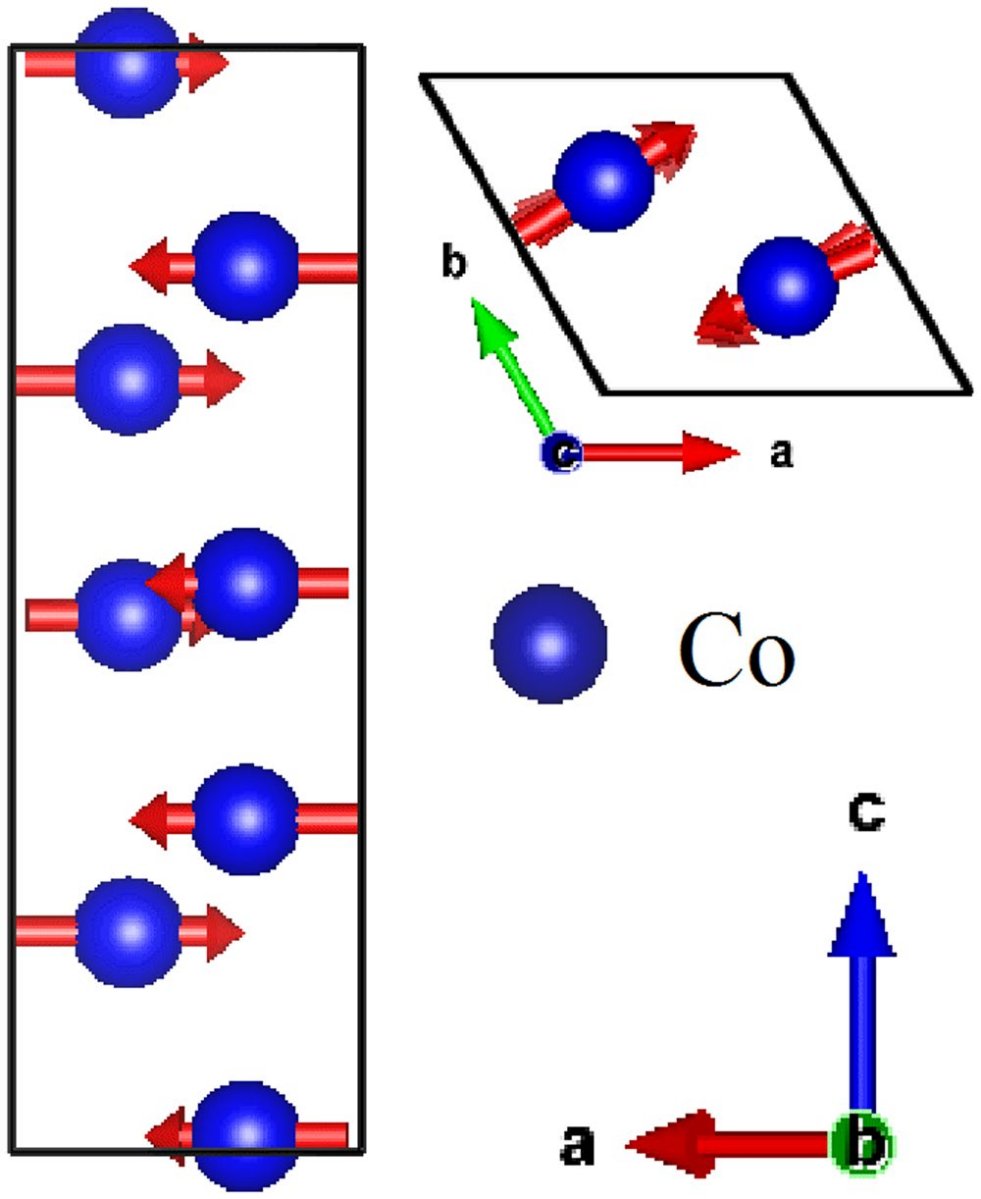
Magnetic structure of Co_4_Nb_2_O_9_, side view along *b* axis and top view along *c* axis.

In our recent research work^11^, we carefully studied the magnetic structure by using neutron powder diffraction and irreducible representation analysis. We found that the magnetic space group is *C2/c’* (the magnetic point group is 2/*m*’) with two independent sites for Co. Spins on the Co atoms are canted in the *ab*-plane and along *c*-axis to form ferromagnetic chains. Two ferromagnetic chains within the unit cell are coupled antiferromagnetically leading to the overall magnetic moments equal to zero. The magnetic moments calculated for each Co site are 2.32(6) and 2.52(8) µ_B,_ respectively. The magnetic structure drawn by VESTA software^12^ (See Fig. 1) to some extent agrees with the magnetic ground state predicted by theoretical calculation by Solovyev *et al*.^9^. This magnetic structure model was successfully used to explain the magnetoelectric coupling effect in CNO^11^.

### Magnetic field control of electric polarization

**Figure 2:**
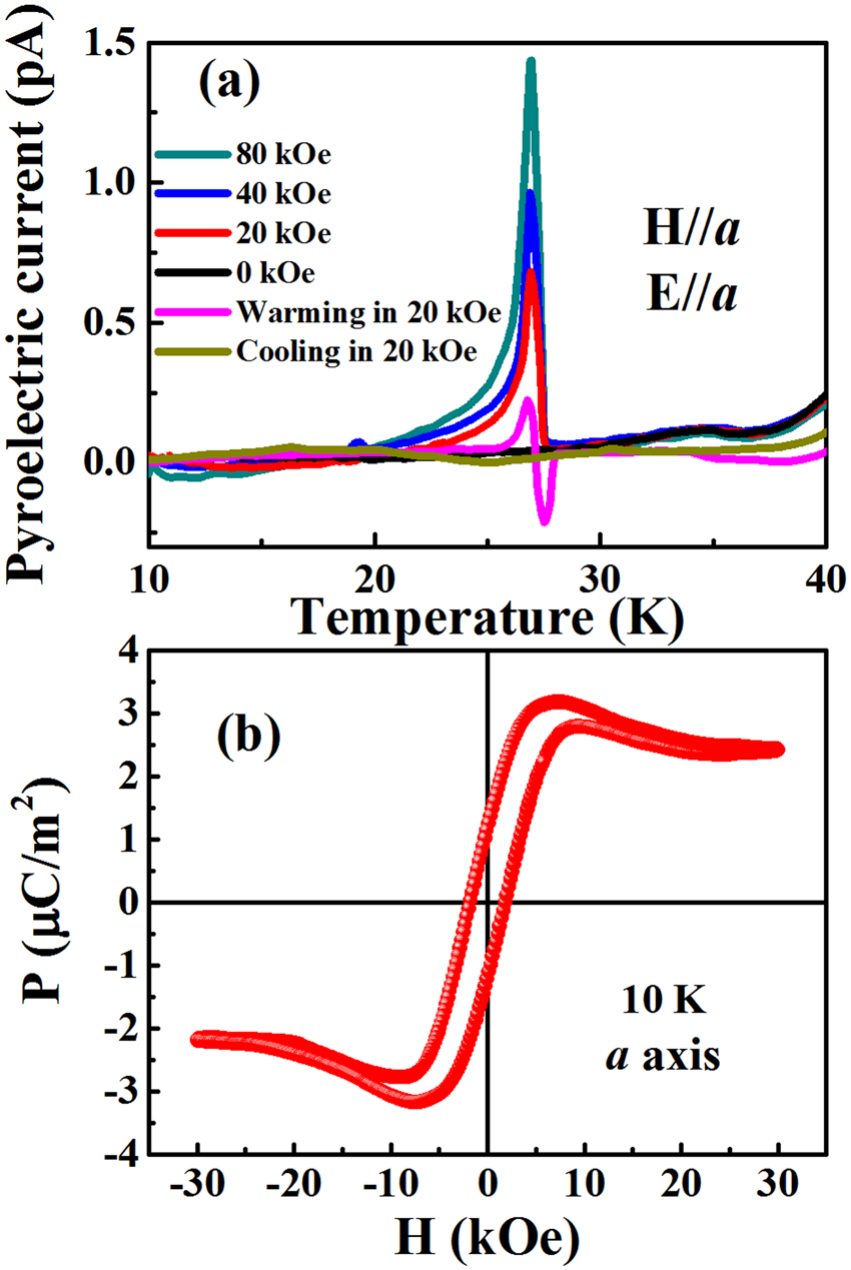
(**a**) The pyroelectric current as a function of temperature under various magnetic fields after ME cooling; (**b**) The polarization as a function of the magnetic field after ME cooling at 10 K.

Prior to the electrical measurements, the sample was first cooled down from 70 to 10 K with an electric field of 1.2 MV/m and a magnetic field of 0, 20, 40 and 80 kOe was applied along the *a*-axis. After ME fields-cooling, the pyroelectric current was recorded with increasing temperature without removing the external magnetic field. As shown in Fig. 2(a), and similarly to Co_4_Nb_2_O_9_ polycrystalline, no anomaly in the pyroelectric current is observed without any applied magnetic field. However, when a magnetic field H is applied, a peak in the pyroelectric current associated with the antiferromagnetic T_N_ temperature of the sample is observed, and the peak amplitude becomes more pronounced with increasing H meaning that the polarization strength depends on the magnetic field. In order to clarify the relationship between the external magnetic field and pyroelectric current response, we carried out further measurements along two other regimes. One corresponds in performing a cooling with an electric field of 1.2 MV/m and a magnetic field of 20 kOe, and measuring pyroelectric current during warming process after removing the external magnetic field at 10 K. In the second one, the sample was first cooled down with an electric field of 1.2 MV/m and zero magnetic field, and then the pyroelectric current was measured during warming process after applying an external magnetic field of 20 kOe at 10 K. As shown in Fig. 2(a), no anomaly in the pyroelectric current is observed for applied external magnetic field during cooling process. By contrast, an anomaly is observed through the zero for applied external magnetic field of 20 kOe during warming process. It is obvious that there is a polarization induced in CNO, and only if, an external magnetic field is applied during heating to make sure the spin flop transition occurs. Note also that the pyroelectric current measured under 20 kOe applied only during heating process, is smaller than that measured with the same magnetic field value but applied during both cooling and heating processes. This means that the polarization in Co_4_Nb_2_O_9_ and therefore the ferroelectricity can be developed below T_N_ only if the magnetic field is kept applied during warming process. It is also worth mentioning that there is a tail in the pyroelectric current below T_N_ which indicates that the depolarization (or destabilization of the polarization) does not take place suddenly but continuously.

In order to further clarify the relationship between polarization P and magnetic field H, P-H curve was calculated by integrating displacement current which was measured as a function of the magnetic field after ME cooling. The polarization could also be reversed by changing the direction of magnetic field as shown in Fig. 2(b). The coercive magnetic field is about 1.81 kOe while the remnant polarization is 1.2 µC/m^2^. It is worth noting that the P-H curve presents an abnormal behavior in the vicinity of 7.5 kOe, which is just correspondent to the critical magnetic field of spin flop as shown in our previous work^7^. Such a consistency sufficiently proves that there indeed exists a nonlinear ME effect in the studied sample, which is originated from the change of spin configuration.

### Electric field control of magnetization

**Figure 3:**
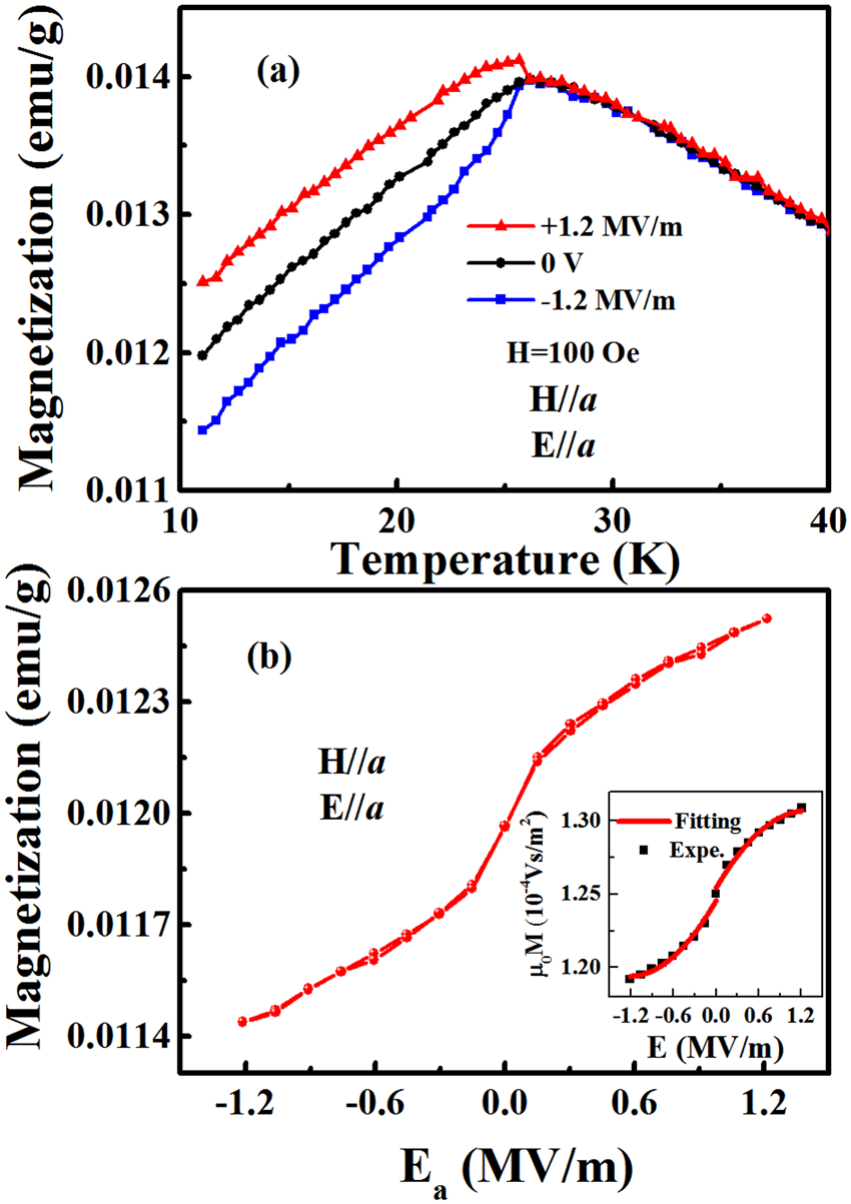
(**a**) The temperature dependence of magnetization under −1.2, 0, and 1.2 MV/m after ME cooling at 100 Oe, 1.2 MV/m, respectively. (**b**) The magnetization as a function electric field after ME cooling at 100 Oe, 1.2 MV/m, respectively.

For the polycrystalline Co_4_Nb_2_O_9_, the effect of electric control of magnetization was reported^5^ in its ferroelectric state (under a cooling magnetic field of 50 kOe). Interestingly here, the control of magnetization by electric field occurs in the paraelectric state for CNO single crystal along the *a*-axis. As shown in Fig. 3(a), the thermomagnetic curves of CNO along *a*-axis are measured at 0.1 kOe and for selected electric field values (−1.2, 0, 1.2 MV/m). In that case, the sample is in its paraelectric state because for such a low magnetic field, the spin flop transition does not occur (see Fig. 3 in ref.^7^). Before these measurements, an ME cooling is performed under 0.1 kOe (H < H_C_ where H_C_ is the critical field for the occurrence of the spin-flop transition, H_C_~7.5 kOe in single crystal and 11 kOe for polycrystalline samples) and 1.2 MV/m in order to ensure the same initial magnetization-polarization state of the sample. As shown in Fig. 3(a), the magnetization versus temperature measured in heating process for different electric field values i.e. 1.2 MV/m, 0 MV/m and −1.2 MV/m show different values of the magnetization below T_N_~27.5 K. Indeed, for temperatures below T_N_, each electric field value gives a different magnetization value. It is clear that the magnetization value increases or decreases of the same magnitude with respect to the magnetization at zero electric field when an electric field, of same amplitude but opposite in sign, is applied. In contrast, the magnetization above T_N_ keeps unchanged with the different applied electric field values, which is ascribed to the disappearance of polarization. It is interesting to stress here that below T_N_ the magnetization can be tuned using a small electric field even under a weak cooling magnetic field. In these low magnetic field conditions, CNO is expected to be in its paraelectric state. However, this state shows non-ergodic behavior as the response of the system depends on the way the state is achieved (here different electric field value gives different magnetization value). At this stage, it is possible to invoke the existence of some clusters which size and/or number might depend on the applied fields.

To study the electric field control of magnetization, the electric field (E) dependence of magnetization (M) along *a*-axis at 10 K was measured. As is shown in Fig. 3(b), there is no hysteresis for M-E curve. Different from previously reported on polycrystalline^5^ and single crystal CNO^10^, the nonlinear M-E curve of the single crystal CNO which implies that CNO is a linear ME material with high order (nonlinear) component. This result is consistent with the P-H curve at 10 K (See Fig. 2(b)). To approximately estimate the converse ME coefficients in the M-E curve, both upper and lower branches were fitted by the formula of ${\mu }_{0}M={\mu }_{0}M(0)+{\alpha }_{e}E+\frac{1}{2}\gamma {E}^{2}$, respectively^13^. The obtained results (see Fig. 3(b)) show that the linear (${\alpha }_{e}$) and quadratic ($\gamma$) coefficients equal to ~8.27 ps/m and ~−6.46 ps/MV for upper branch, as well as for the lower branch, the ${\alpha }_{e}$ and $\gamma$ equal to ~8.38 ps/m and ~6.75 ps/MV. Clearly, the linear coefficients obtained by two branches are almost equivalent, while the quadratic coefficients obtained by two branches are just the opposite. The latter one can be attributed to the change of electric field direction.

The magnetic space group is *C2/c’* while the magnetic point group is 2/*m*’ for CNO. Actually, for the magnetic point group 2/*m*’, which is the zero-field magnetic structure of CNO, it has no quadratic term for ME coupling. However, for the mixed magnetic phase under magnetic field or electric field (due to magnetoelectric effect). For example, under electric field, the two polarizations (up and down) will be effected by the electric field. The response from the up one will increase while the other one will suppress or even change (flip) the direction. This would cause the change of magnetic structure due to the formula $P={e}_{ij}\times ({S}_{i}\times {S}_{j})$ in a reverse way. The final effect is the magnetic moments have to reorient to allow this polarization to take place. Due to such changes, the new phase could be a mixture of 2/m and 2/m’. For the ferromagnetic phase 2/m, the tensor can be described by form (1), which allows the nonlinear HEE term in the thermodynamic potential formula ${\mu }_{0}M={\mu }_{0}M(0)+{\alpha }_{e}E+\frac{1}{2}\gamma {E}^{2}$, where $\frac{1}{2}\gamma {E}^{2}$ is the nonlinear term.

1 $$[\begin{array}{ccc}0 & 0 & 0\\ {\gamma }_{24} & {\gamma }_{24} & {\gamma }_{24}\\ 0 & 0 & 0\end{array}\,\begin{array}{ccc}{\gamma }_{14} & 0 & {\gamma }_{16}\\ 0 & {\gamma }_{25} & 0\\ {\gamma }_{34} & 0 & {\gamma }_{36}\end{array}]$$

Based on the above discussion, the nonlinear magnetoelectric effect could be explained by existence of high order component in the studied sample. In this case, the ferromagnetic phase 2/*m* has quadratic term for ME coupling. For this speculation, more work, such as neutron diffraction under magnetic or electric field, is needed to confirm it.

**Figure 4:**
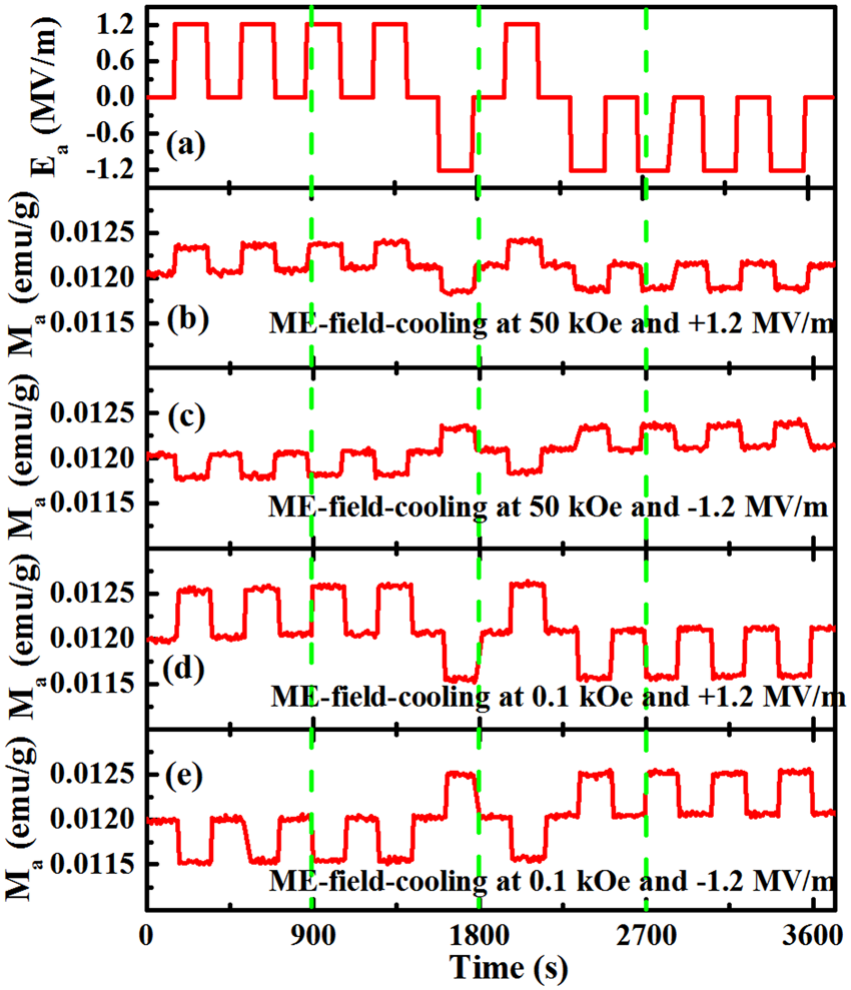
Temporal evolution of magnetization along *a* (M_*a*_) responding to periodically applied electric fields along *a* (E_*a*_) at T = 10 K and H = 50 kOe and 0.1 kOe, respectively. (**a**) shows applied E_*a*_ as a function of time. (**b**,**c**,**d** and **e**) display the corresponding M_*a*_ data.

To further investigate the responses of magnetization to the electric field, as shown in Fig. 4(a), the variation of magnetization as a function of a periodic electric field is measured. Figure 4(b,c,d,e) show the corresponding magnetic responses. The magnetization decreases or increases as the same or different sign of the applied electric fields, respectively. More interestingly, a pronounced response was observed under a small cooling magnetic field which cannot even cause the spin flop. This electric field control of magnetism in the paraelectric state would open up a promising spark to implementing and testing the electric field control of magnetism in other high-temperature multiferroics. Furthermore, the responses may find potential uses in magnetic data storage and switching devices such as nonvolatile magnetic memory which facilitates three distinct states of magnetization.

**Figure 5:**
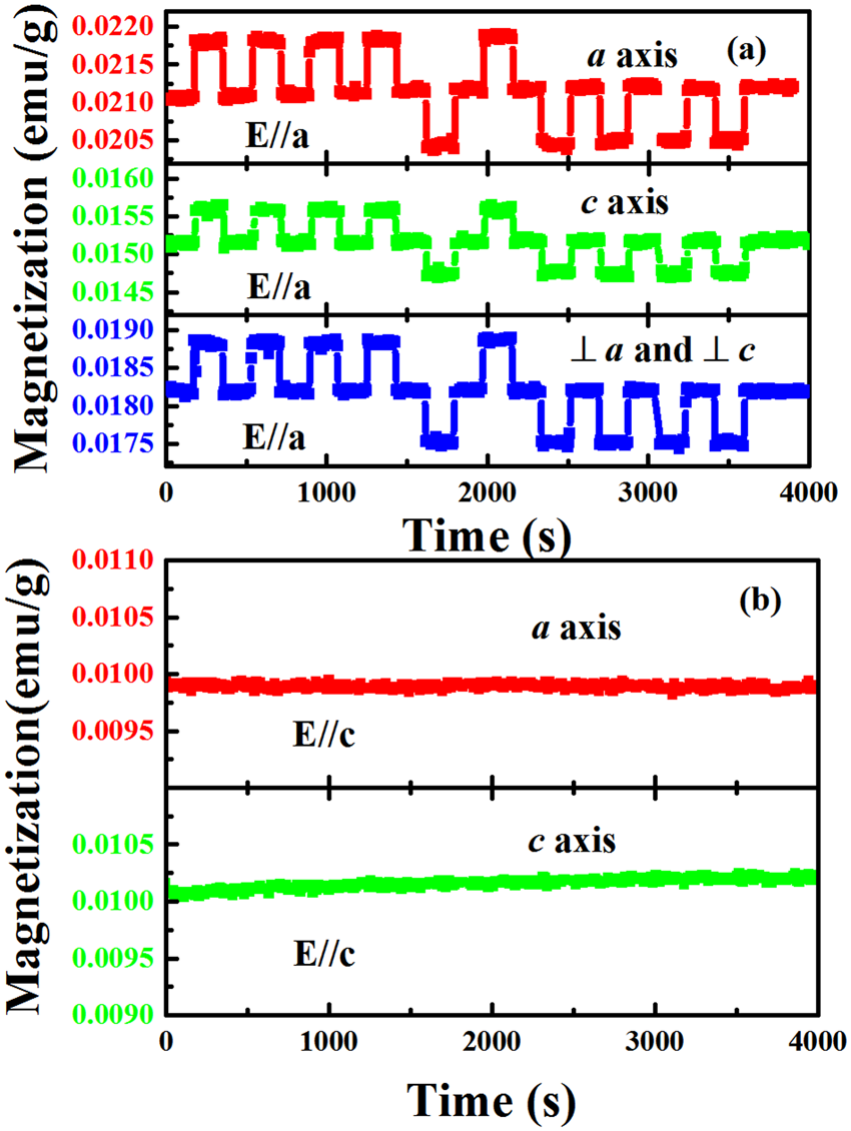
(**a**) Temporal evolution of magnetization along *a*, *c* and the axis orthogonal with *a* and *c* responding to periodically applied electric fields along *a* (E_*a*_) at T = 10 K and H = 0.1 kOe. (**b**) Temporal evolution of magnetization along *a* and *c* axis responding to periodically applied electric fields along *c* (E_*c*_) at T = 10 K and H = 0.1 kOe.

In order to clarify the directional relationship between magnetic response and applied electric field, the magnetic responses along different axes under *a* and *c* direction electric field were measured and shown in Fig. 5(a) and (b). It is obvious that the magnetization of every axis responds to electric field applied along *a*-axis, but fails to do so when the electric field is applied *c*-axis. The magnetic response under different directional electric field makes it possible for us to study the interaction between the electric field and the spin. As we know, the antiparallel Co^2+^ spins locate in *ab* plane, *a*-axis electric field will yield a net electric field along the spin but *c* axis electric field could only be perpendicular to the spin.

## Discussion

According to our recent explanation to the ME coupling mechanism in CNO^11^, the magnetic-field-induced electrical polarization is along the [110] direction due to the spin-current effect^14^. Reversely, if applying electric fields in any direction in the *ab* plane, the electric fields should induce variations of magnetization in the corresponding directions. However, this effect will not exist along the *c* axis because the electrical polarization along the *c* axis is always zero. Therefore, the observed magnetization response under electric fields in this study is understandable on the basis of our ME model^11^. At the same time, the systematic electrical and magnetic measurement provides new evidence about the spin-current mechanism of the ME in CNO proposed in our recent work^11^. The ME measurement by Khanh *et al*.^8^ indicates the ME effect along the *c* axis, which is contradictive to the above discussion and could be due to the slight misalignment of the sample in the measurement. Similar problem was observed in the earlier work of Cr_2_O_3_^15^.

In summary, we have studied the detailed magnetoelectric (ME), magnetic and electric control of magnetic properties in CNO crystal. A detailed ME measurement reveals a nonlinear ME effect, whose linear (${\alpha }_{e}$) and quadratic ($\gamma$) coefficients equal to ~8.27 ps/m and ~−6.46 ps/MV for upper branch, as well as for the lower branch, the ${\alpha }_{e}$ and $\gamma$ equal to ~8.38 ps/m and ~6.75 ps/MV. The $\gamma$ change of sign can be attributed to the change of electric field direction. A pronounced response was observed under a small cooling magnetic field which cannot even cause the spin flop. Furthermore, we also found that the magnetization of every axis responds to electric field *a*pplied along *a*-axis, but fails to do so when the electric field is applied *c*-axis. These findings further indicate that the spin-current mechanism is the nature of the ME effect in CNO single crystal.

## Methods

A single-crystal specimen of Co_4_Nb_2_O_9_ was grown in an optical floating zone furnace. The same sample was investigated in our previous work^7^. Magnetic properties were measured by a Physical Property Measurement System (PPMS-9, Quantum Design Inc.) with VSM (Vibrating Sample Magnetometer) option. To measure magnetization as a function of electric field, a homemade insert which allows the application of electric field was installed in the magnetometer. The plates with the large [100] and [001] plane of 2 * 2 mm^2^ were sliced out from the crystal rod, and both the end [100] and [001] surfaces were painted with silver paste as the electrodes. The pyroelectric current was collected using an electrometer (Keithley 6517B) after poling the sample in an electric and magnetic field. The electric polarization as a function of temperature and magnetic field was calculated by integrating displacement current with time. Specifically, the specimen was cooled down to 10 K while applying a poling electric field of 1.2 MV/m along [100] direction. In order to release any charges accumulated on the sample surfaces or inside the sample, the sample was short-circuited for a long-enough time (1 h). During the recording of pyroelectric current, the sample was heated slowly at a warming rate of 2 K/min. Note that the magnetic field was applied throughout the cooling and warming processes.
